# Analyzing the pathogenesis of systemic lupus erythematosus complicated by atherosclerosis using transcriptome data

**DOI:** 10.3389/fimmu.2022.935545

**Published:** 2022-07-22

**Authors:** Yimin Wang, Wenge Su, Yunlun Li, Jie Yuan, Minghao Yao, Xiaoyi Su, Yifei Wang

**Affiliations:** The First Clinical Medical College, Shandong University of Traditional Chinese Medicine, Jinan, China

**Keywords:** systemic lupus erythematosus, atherosclerosis, bioinformatics, differentially expressed genes, hub genes, immune cell infiltration

## Abstract

**Background:**

Accumulating evidence supports the predisposition of systemic lupus erythematosus (SLE) to atherosclerosis (AS). However, the common pathogenesis of these two diseases remains unclear. This study aimed to explore the mechanisms of SLE complicated by AS.

**Methods:**

Gene expression profiles of SLE (GSE50772) and AS (GSE100927) were downloaded from the Gene Expression Omnibus. We analyzed differentially expressed genes (DEGs) of SLE and AS and performed enrichment analyses separately. After analyzing the common DEGs (CDEGs), we performed functional enrichment analysis, protein-protein interaction (PPI) network analysis, and hub genes (HGs) identification of CDEGs. Then, we performed a co-expression analysis of HGs and verified their expression and diagnostic value. We further explored immune cell infiltration and analyzed the correlation between HGs and infiltrating immune cells (IICs). Finally, we verified the reliability of the screening pathway.

**Results:**

We obtained 530 DEGs from the GSE50772 dataset and 448 DEGs from the GSE100927 dataset. The results of the enrichment analysis showed that there were many similar immune- and inflammation-related processes between the two diseases. We analyzed 26 CDEGs (two downregulated genes and 24 upregulated genes) and enrichment analysis highlighted the important role of the IL-17 signaling pathway. We identified five HGs (*CCR1*, *CD163*, *IL1RN*, *MMP9*, and *SIGLEC1*) using the CytoHubba plugin and HGs validation showed that the five HGs screened were reliable. Co-expression networks showed that five HGs can affect mononuclear cell migration. Immune cell infiltration analysis indicated monocytes in SLE and M0 macrophages in AS accounted for a high proportion of all IICs, and the difference in infiltration was obvious. We also found a significant positive correlation between *CCR1*, *CD163*, *IL1RN*, and *MMP9* and monocytes in SLE, and a significant positive correlation between *CCR1*, *IL1RN*, *MMP9*, and *SIGLEC1* and M0 macrophages in AS. Pathway validation also demonstrated that the IL-17 signaling pathway was a key pathway for the differentiation of monocytes into macrophages.

**Conclusions:**

The five HGs may promote the differentiation of monocytes into macrophages by influencing the IL-17 signaling pathway, leading to SLE complicated by AS. Our study provides insights into the mechanisms of SLE complicated by AS.

## Introduction

Systemic lupus erythematosus (SLE) is an autoimmune disease characterized by alternating episodes and remissions, and can cause damage to multiple organs in the body. Previous studies have demonstrated that SLE can increase the relative risk of atherosclerosis (AS) (1). Recent data suggest that approximately 7% of SLE patients experience a cardiovascular event, and that the risk of cardiovascular disease (CVD)-related death increases approximately two to threefold in SLE patients compared to the general population (2, 3).

Although SLE is considered a risk factor for AS, the common pathogenesis of these two diseases remains unclear. In addition to the common traditional risk factors associated with AS, specific risk factors for the immune and inflammatory profiles of SLE may be more important for worsening AS (4). Abnormal immune status and energy metabolism in SLE patients lead to strong oxidative stress. Molecular targets such as proinflammatory high-density lipoprotein (HDL) and oxidized lipids play an important role in accelerating SLE complicated by AS (5). Pro-inflammatory cytokines also play an important role in accelerating SLE complicated by AS. Interferon (IFN)-α can directly enhance the expression of chemokines and adhesion molecules and upregulate the expression of type A scavenger receptors in macrophages, promoting the formation of macrophage-derived foam cells (6). Macrophage migration inhibitor (MIF) not only promotes low-density lipoprotein (LDL) uptake and leads to plaque formation but also increases the expression of matrix metallopeptidase (MMP)-1 and MMP-9 to induce plaque rupture (7, 8). In addition, changes in immune cells, such as abnormal T cell formation (9), neutrophil extracellular trap (NET) formation (10) and B cell activation (11) also play an important role in the pathogenesis of SLE complicated by AS. Currently, AS remains one of the main causes of death in patients with advanced SLE. It is of great significance to analyze the pathogenesis of SLE complicated by AS to find key therapeutic targets and prolong the life of SLE patients.

We aimed to explore the common pathogenesis of these two diseases, based on the common transcriptional characteristics of SLE and AS. We analyzed two datasets (GSE50772 and GSE100927) and obtained the common differentially expressed genes (CDEGs) in SLE and AS. we performed functional enrichment analysis, protein-protein interaction (PPI) network analysis, and hub genes (HGs) identification of CDEGs. Then, we performed a co-expression analysis of HGs and verified their expression and diagnostic value. We further explored immune cell infiltration and analyzed the correlation between HGs and infiltrating immune cells (IICs). Finally, we verified the reliability of the screening pathway. The results of our study provide insights into the pathogenesis of SLE complicated by AS.

## Materials and methods

### Data source

We downloaded the relevant microarray datasets from the Gene Expression Omnibus (http://www.ncbi.nlm.nih.gov/geo). GSE50772 (12) contains the expression data of peripheral blood mononuclear cells (PBMC) from 61 patients with SLE and 20 normal controls. GSE100927 (13) contains the expression data of 69 AS plaque tissue samples and 35 control samples.

### Identification of DEGs

According to the probe annotation file, probe names in each data set were converted to gene names, DEGs were filtered using the “limma” package in R (version 4.1.2), the volcano diagram of DEGs was drawn using the “ggplot2” package, and DEGs with | log2fold change (FC) | > 1 and *p* < 0.05 were considered statistically significant. The DEGs obtained by SLE and AS were intersected to obtain the CDEGs.

### Enrichment analyses of DEGs

DEGs were imported into the Metascape (14) online analysis platform (https://metascape.org/gp/index.html) for enrichment analysis using default parameters. In addition, we also used the “clusterProfiler” package for Kyoto Encyclopedia of Genes and Genomes (KEGG) analysis.

### Selection and analysis of HGs

CDEGs were imported into the STRING (http://string-db.org) database (15) to build a PPI network (interaction combined score was > 0.4) and visualize the network in Cytoscape (version 3.8.0). CytoHubba (16) is a Cytoscape plugin that includes 12 algorithms. We found that 12 algorithms can effectively identify HGs in this study, so we randomly selected five algorithms and used the intersection results to identify HGs. The co-expression network of the HGs was constructed using GeneMANIA (http://www.genemania.org/).

### Validation of HGs and screened pathways in other data sets

We downloaded the GSE81622 (17) and GSE43292 (18) datasets for HGs expression (*p* < 0.05) and diagnostic value verification. GSE81622 contains PBMC expression data from 25 patients with SLE and 30 normal controls. GSE43292 contains the expression data of 32 AS plaque tissue samples and 32 control samples. We downloaded the GSE37356 (19) datasets to verify the screened pathways. GSE37356 contains inflammatory expression profiles in monocyte to macrophage differentiation amongst 20 patients with SLE and 16 healthy controls with and without an AS phenotype.

### Evaluation of immune cell infiltration and correlation analysis between HGs and IICs

We used the CIBERSORT algorithm to analyze GSE50772 and GSE100927 gene expression data (*p* < 0.05), and the “corrplot” and “ggplot2” packages to draw the related heatmap and violin diagram of the IICs. The “ggstatsplot” package was used to analyze the Spearman correlation and the “ggplot2” package was used to visualize the results.

## Results

### Identification of DEGs

**Figure 1:**
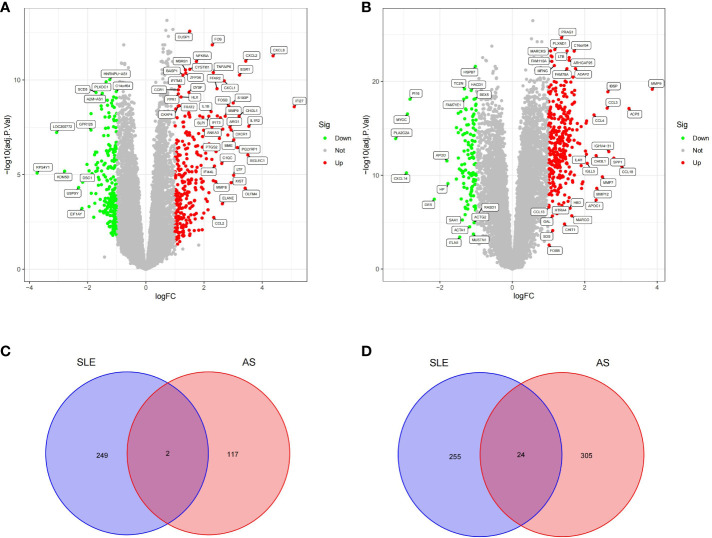
Volcano plot and Venn diagram. **(A)** Volcano plot of differentially expressed genes (DEGs) in GSE50772. **(B)** Volcano plot of differentially expressed genes (DEGs) in GSE100927.The up-regulated genes were marked with red, the down-regulated genes were marked with green, and the genes with no significant changes were marked with gray. **(C)** Venn diagram of down-regulated genes in GSE50772 and GSE100927. **(D)** Venn diagram of up-regulated genes in GSE50772 and GSE100927.

A total of 530 DEGs (**Supplementary File 1**) were obtained from the GSE50772 dataset, of which 251 DEGs were downregulated and 279 DEGs were upregulated (**Figure 1A**); 448 DEGs (**Supplementary File 2**) were obtained from the GSE100927 dataset, of which 119 DEGs were downregulated and 329 DEGs were upregulated (**Figure 1B**). We intersected downregulated and upregulated DEGs from the two data sets, and obtained 26 CDEGs (**Supplementary File 3**) (**Figures 1C, D** **)** with the same expression trend.

### Analysis of the functional characteristics of DEGs

**Figure 2:**
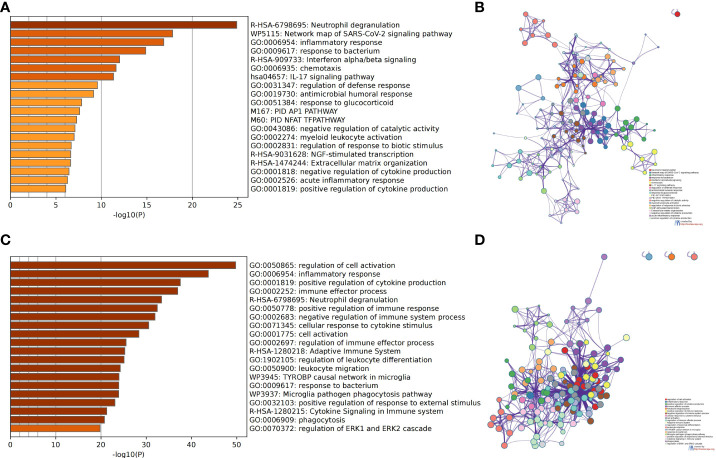
Analysis of functional characteristics of DEGs. **(A)** Bar chart of the DEGs in GSE50772 functional enrichment analysis. **(B)** Network diagram of the DEGs in GSE50772 functional enrichment analysis. **(C)** Bar chart of the DEGs in GSE100927 functional enrichment analysis. **(D)** Network diagram of the DEGs in GSE100927 functional enrichment analysis.

We used the Metascape analysis platform for the enrichment analysis of DEGs in GSE50772 and GSE100927. We found that neutrophil degranulation, inflammatory response, interferon alpha/beta signaling, and the IL-17 signaling pathway were significantly enriched in SLE (**Figures 2A, B** **)**. We also found that regulation of cell activation, inflammatory response, positive regulation of cytokine production, immune effector process, and neutrophil degranulation were significantly enriched in AS (**Figures 2C, D** **)**. Many immune- and inflammation-related processes, such as neutrophil degranulation and the inflammatory response, play an important role in SLE and AS.

**Figure 3:**
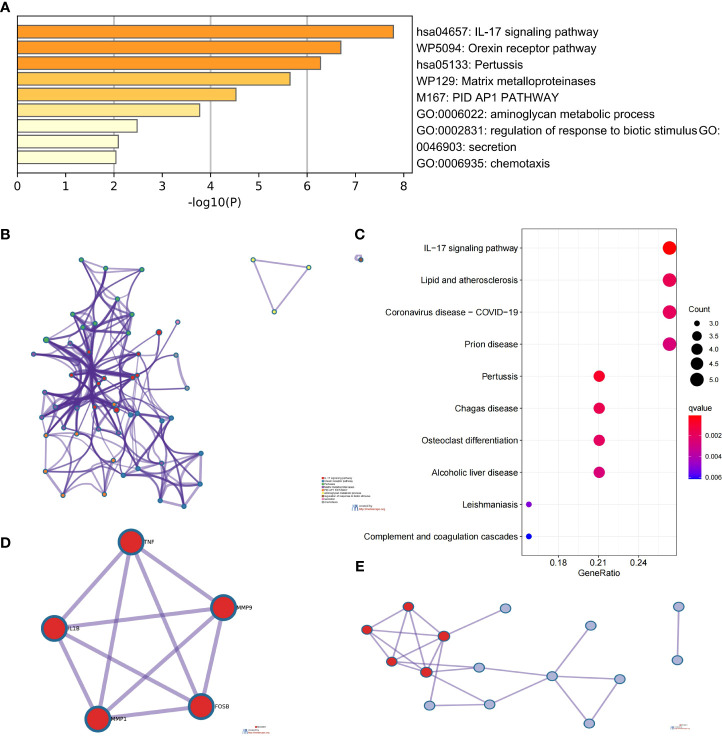
Analysis of functional characteristics of common DEGs (CDEGs). **(A)** Bar chart of the CDEG functional enrichment analysis. **(B)** Network diagram of the CDEG functional enrichment analysis. **(C)** Results of the Kyoto Encyclopedia of Genes and Genomes **(**KEGG) enrichment analysis of CDEGs. **(D)** MCODE1 component identified in CDEGs. **(E)** Protein-protein interaction (PPI) network identified in CDEGs; red represents MCODE1.

We used the Metascape analysis platform for the enrichment analysis of CDEGs and found that the IL-17 signaling pathway, orexin receptor pathway, matrix metalloproteinases, and regulation of response to biotic stimulus were significantly enriched (**Figures 3A, B** **)**. The results of KEGG enrichment analysis were related to the IL-17 signaling pathway and lipid and atherosclerosis (**Figure 3C**). The molecular complex detection (MCODE) algorithm provided by Metascape analyzed a gene module (MCODE1) (**Figures 3D, E** **)** that contains five genes (*TNF*, *MMP9*, *FOSB*, *MMP1*, and *IL1B*). The results of functional enrichment analysis were related to the IL-17 signaling pathway, interleukin-4 and interleukin-13 signaling, and photodynamic therapy-induced NF-κB survival signaling (**Table 1**). Thus, the IL-17 signaling pathway may be a key mechanism in SLE complicated by AS.

**Table 1:** Gene ontology (GO) enrichment analysis of genes in MCODE1.

MCODE	GO	Description	Log10 (P)
MCODE1	hsa04657	IL-17 signaling pathway	-12.6
MCODE1	WP3617	Photodynamic therapy-induced NF-kB survival signaling	-11.1
MCODE1	R-HSA-6785807	Interleukin-4 and Interleukin-13 signaling	-9.1

### PPI network construction and selection and analysis of HGs

**Figure 4:**
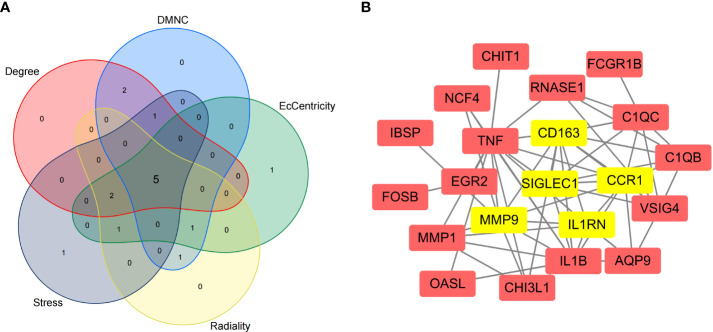
Selection of hub genes (HGs). **(A)** Venn diagram of the results screened by five algorithms of CytoHubba. **(B)** Location of the five HGs in the PPI network.

**Figure 5:**
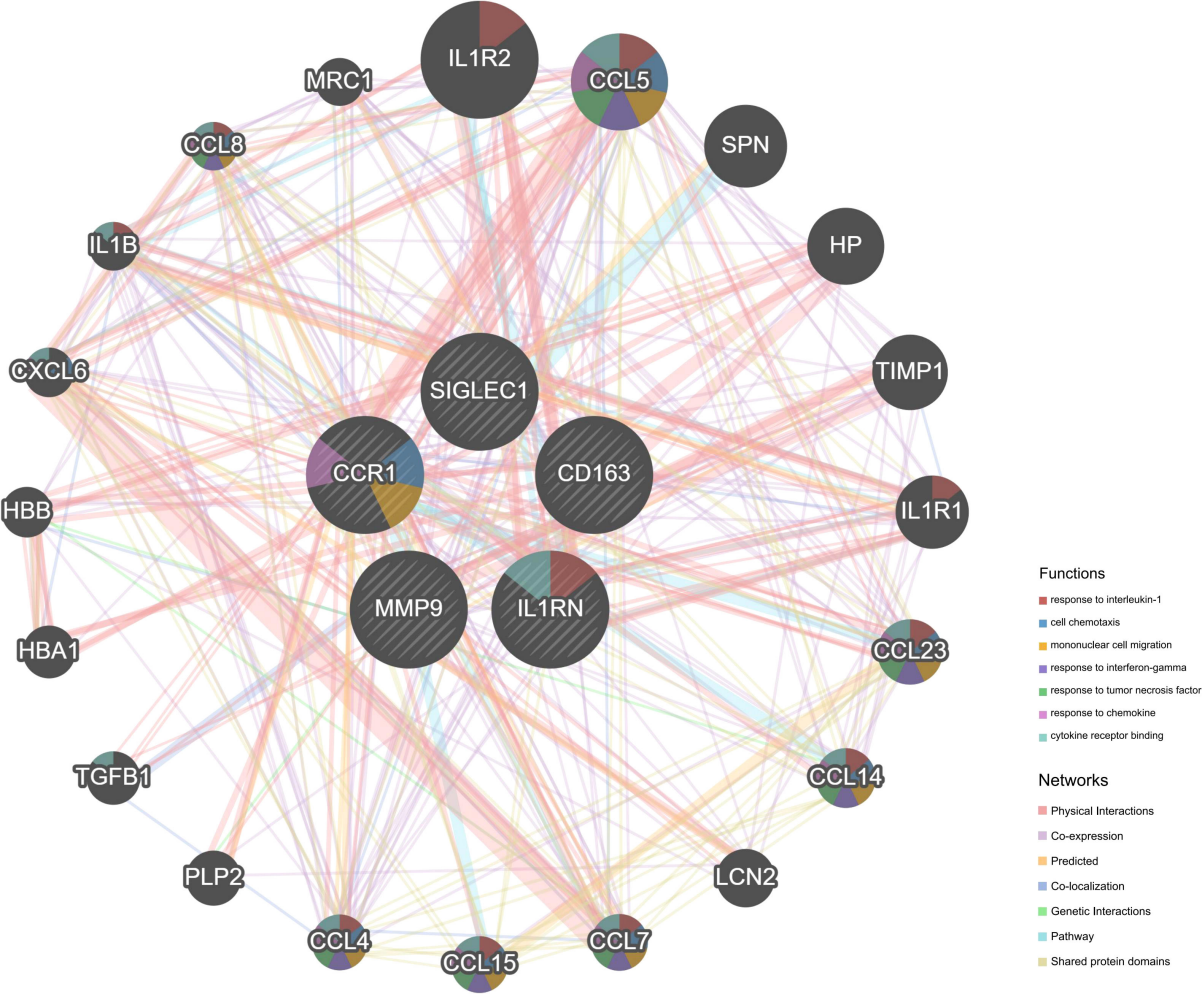
Five HGs and their co-expression genes analyzed using GeneMANIA.

We imported the CDEGs into the STRING database and built a PPI network. Five algorithms (degree, DMNC, eccentricity, radiality, and stress) in the CytoHubba plugin were used to identify HGs. **Table 2** lists the top 10 genes screened for using the five algorithms. We took the intersection of the screening results of the five algorithms and determined five HGs (*CCR1*, *CD163*, *IL1RN*, *MMP9*, and *SIGLEC1*) (**Figure 4A**). **Figure 4B** shows the locations of the five HGs in the PPI network. To further reveal the biological characteristics of HGs, we constructed and analyzed a network of HGs and their co-expressed genes (**Figure 5**) based on GeneMANIA. Five HGs showed a complex PPI network: physical interaction, 77.64%; total expression, 8.01%; prediction, 5.37%; total location, 3.63%; genetic interaction, 2.87%; pathway, 1.88%; and shared protein domains, 0.60%. The biological function of HGs is related to immune and inflammatory-related processes, such as response to interleukin-1, response to tumor necrosis factor, cell chemotaxis, mononuclear cell migration, response to interferon-gamma, and response to chemokines.

**Table 2:** The top 10 HGs as ranked in CytoHubba.

Degree	DMNC	EcCentricity	Radiality	Stress
C1QB	C1QB	CCR1	CCR1	C1QC
C1QC	C1QC	CD163	CD163	CCR1
CCR1	CCR1	CHI3L1	CHI3L1	CD163
CD163	CD163	IL1B	IL1B	EGR2
IL1B	CHI3L1	IL1RN	IL1RN	IL1B
IL1RN	IL1RN	MMP9	MMP1	IL1RN
MMP9	MMP1	NCF4	MMP9	MMP9
SIGLEC1	MMP9	RNASE1	RNASE1	RNASE1
TNF	SIGLEC1	SIGLEC1	SIGLEC1	SIGLEC1
VSIG4	VSIG4	TNF	TNF	TNF

### Validation of HGs expression and diagnostic value

**Figure 6:**
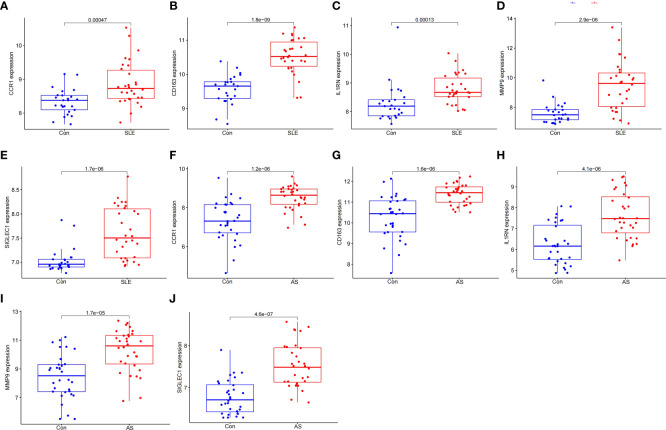
Validation of HGs expression. **(A–E)** Expression of the five HGs verified in GSE81622. **(F–J)** Expression of the five HGs verified in GSE43292.

**Figure 7:**
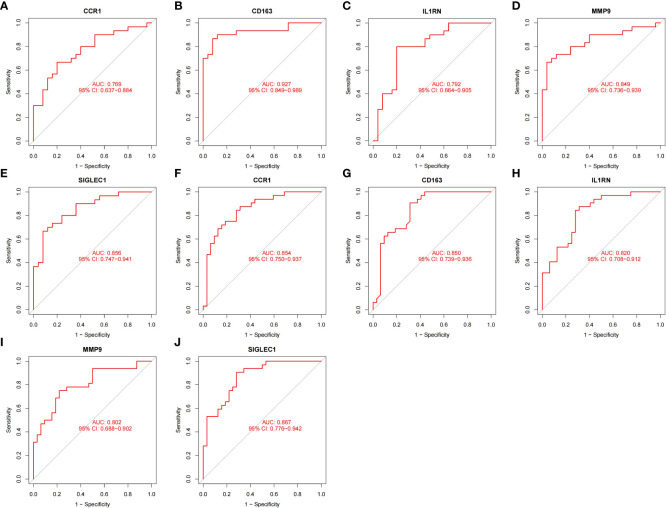
Validation of diagnostic value. **(A–E)** Receiver operating characteristic curve of five HGs in GSE81622. **(F–J)** Receiver operating characteristic curve of five HGs in GSE43292.

To prove the reliability of the selected HGs, the expression of five HGs was verified using GSE81622 and GSE43292 datasets. The results showed that the expression of five HGs in SLE was higher than that in control samples (**Figures 6A–E**) and the expression of five HGs in AS plaques was also higher than that in control samples (**Figures 6F–J**). The receiver operating characteristic curves of the five HGs is shown, and they showed good diagnostic value in SLE (**Figures 7A–E**) and AS (**Figures 7F–J**). This shows that the five HGs screened were reliable.

### Immune cell infiltration results

**Figure 8:**
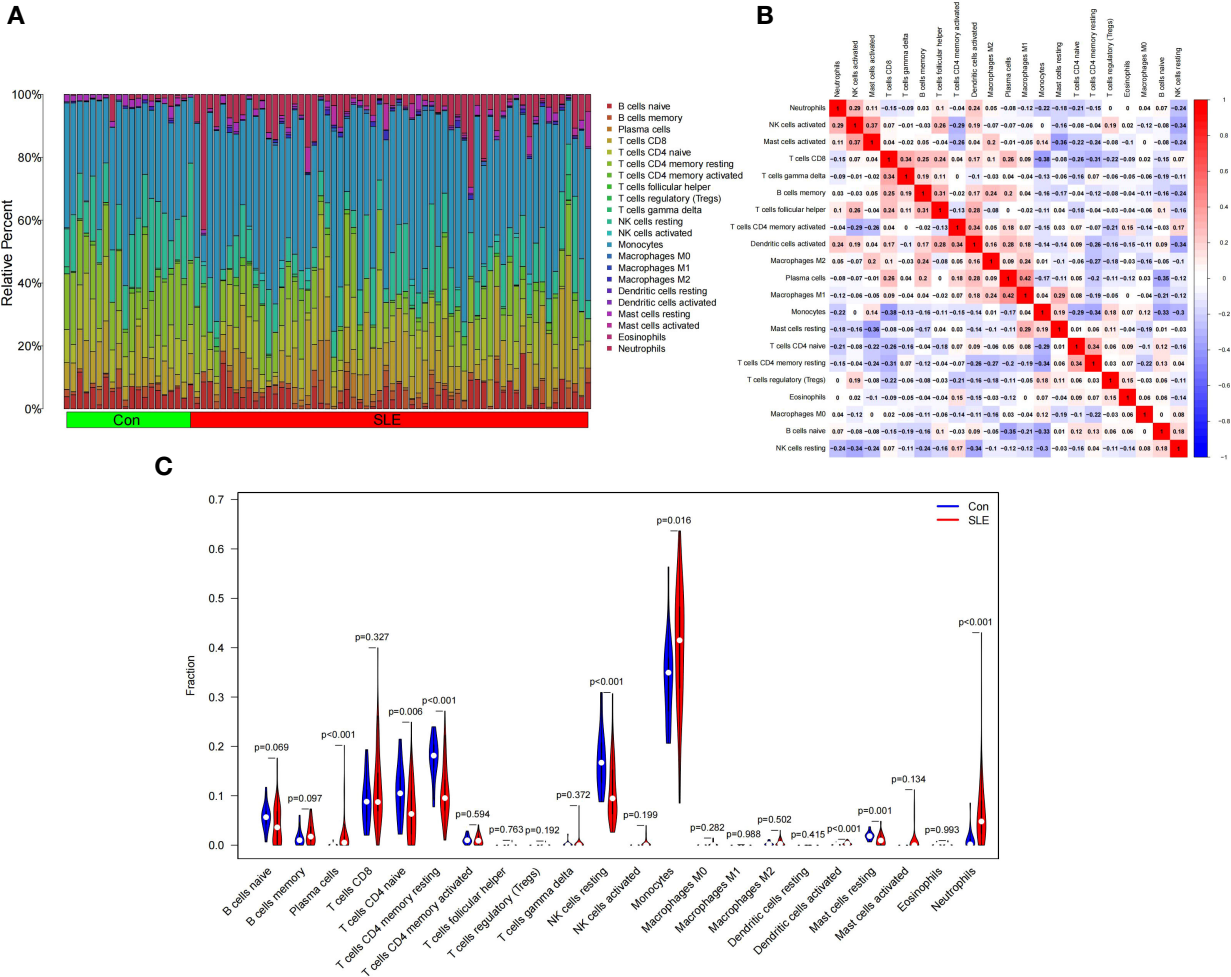
Landscape map of infiltrating immune cells (IICs) in the Systemic Lupus Erythematosus (SLE) and Con groups. **(A)** The relative proportion of IICs in SLE and Con. **(B)** Correlation heatmap between IICs. Red represents positive correlation, blue represents negative correlation, and the number in the square represents correlation. **(C)** Violin diagram showing the difference in immune cell infiltration between SLE and Con. (SLE group shown in red, Con group shown in blue, *p* < 0.05 was considered statistically significant).

**Figure 8A** shows the composition of immune cells in the SLE group and control group. We found that the number of plasma cells, monocytes, activated dendritic cells, and neutrophils in the SLE group were significantly higher than that in the control (*p* < 0.05) and monocytes had the highest proportion of all IICs. The number of CD4 naive T cells, CD4 memory resting T cells, resting NK cells, and resting Mast cells were significantly lower than that in the control (*p* < 0.05) (**Figure 8C**). We also drew a correlation heatmap of IICs (**Figure 8B**). We found that monocytes were negatively correlated with CD8 T cells (*r* = -0.38) and there was a weak correlation among the other IICs.

**Figure 9:**
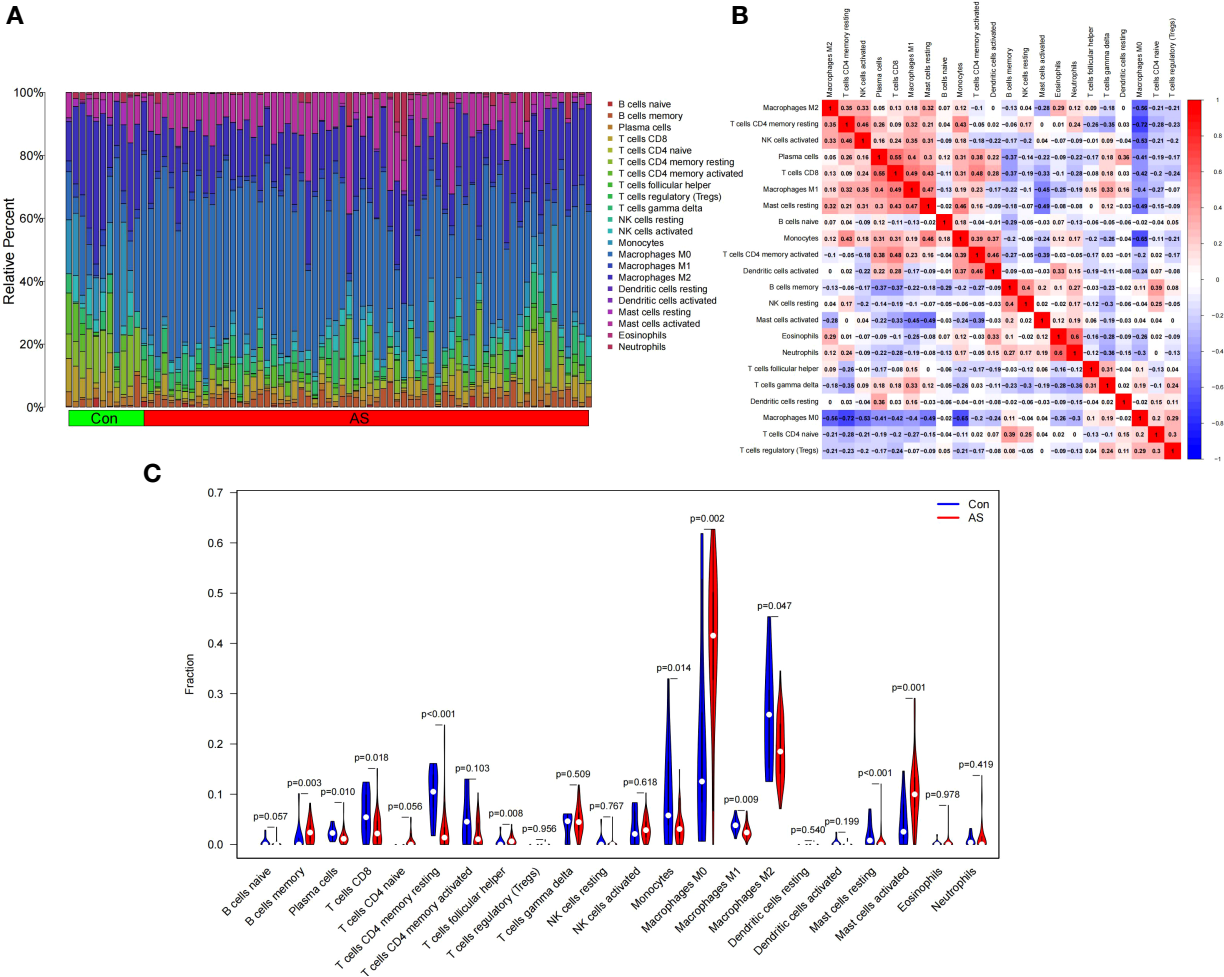
Landscape map of infiltrating immune cells (IICs) in the atherosclerosis (AS) and control (Con) groups. **(A)** The relative proportion of IICs in AS and Con. **(B)** Correlation heatmap between IICs. Red represents positive correlation, blue represents negative correlation, and the number in the square represents correlation. **(C)** Violin diagram showing the difference in immune cell infiltration between AS and Con. (AS group shown in red, Con group shown in blue, *p* < 0.05 was considered statistically significant).

**Figure 9A** shows the composition of immune cells in the AS plaque tissue and control group. We found that the memory of B cells, follicular helper T cells, M0 macrophages, and activated mast cells in atherosclerotic plaques were significantly higher than that in the control tissues (*p* < 0.05) and M0 macrophages had the highest proportion of all IICs. The number of plasma cells, CD8 T cells, CD4 memory resting T cells, monocytes, M1 macrophages, M2 macrophages, and resting mast cells were significantly lower than that in the control tissue (*p* < 0.05) (**Figure 9C**). The correlation heatmap of IICs (**Figure 9B**) show that M0 macrophages were negatively correlated with memory resting CD4 T cells (*r* = -0.72), monocytes (*r* = -0.65), M2 macrophages (*r* = -0.56), activated NK cells (*r* = -0.53), and resting mast cells (*r* = -0.49). Activated mast cells were negatively correlated with M1 macrophages (*r* = -0.45) and resting mast cells (*r* = -0.49). CD8 T cells were positively correlated with plasma cells (*r* = 0.55), M1 macrophages (*r* = 0.49), and resting mast cells (*r* = 0.43).

### Correlation analysis between HGs and IICs

**Figure 10:**
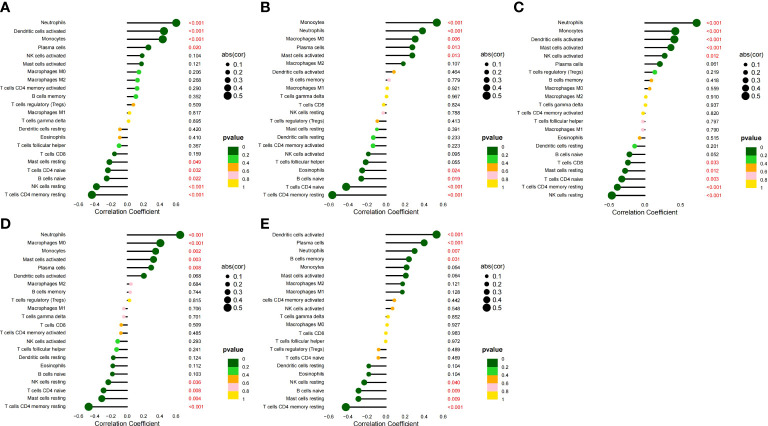
Correlation between five HGs and IICs in SLE. **(A)** Correlation between *CCR1* and IICs. **(B)** Correlation between *CD163* and IICs. **(C)** Correlation between *IL1RN* and IICs. **(D)** Correlation between *MMP9* and IICs. **(E)** Correlation between *SIGLEC1* and IICs.

**Figure 11:**
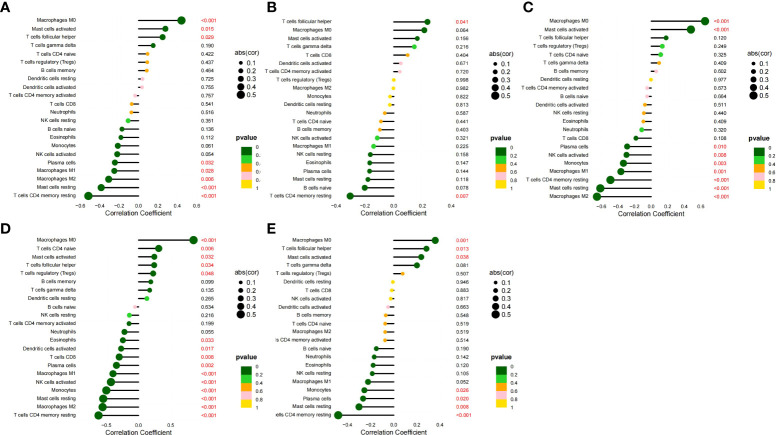
Correlation between five HGs and IICs in AS. **(A)** Correlation between *CCR1* and IICs. **(B)** Correlation between *CD163* and IICs. **(C)** Correlation between *IL1RN* and IICs. **(D)** Correlation between *MMP9* and IICs. **(E)** Correlation between *SIGLEC1* and IICs.

We analyzed the correlation between the five HGs and IICs in SLE (**Figures 10A–E**). We found a significant positive correlation between *CCR1*, *CD163*, *IL1RN*, and *MMP9* and monocytes, and neutrophils (*p* < 0.05), and found a significant positive correlation between *SIGLEC1* and neutrophils (*p* = 0.007). We also analyzed the correlation between the five HGs and IICs in AS (**Figures 11A–E**). We found a significant positive correlation between *CCR1*, *IL1RN*, *MMP9*, and *SIGLEC1* and M0 macrophages, and activated mast cells (*p* < 0.05), and found a significant positive correlation between *CD163* and follicular helper T cells (*p* = 0.041). Thus, the five HGs may play an important role in the pathogenesis of SLE complicated by AS by affecting immune cell infiltration. Monocytes and macrophages may be targets of the five HGs.

### Validation of screened pathways

**Figure 12:**
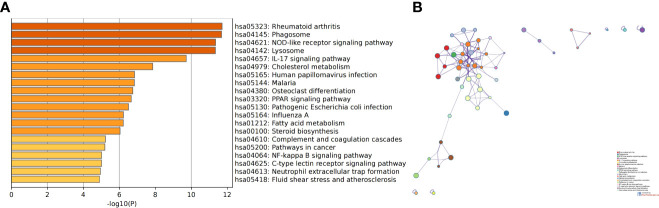
The results of KEGG enrichment analysis of DEGs in GSE37356. **(A)** Bar chart of the KEGG enrichment analysis. **(B)** Network diagram of the KEGG enrichment analysis.

We screened the gene expression profiles of monocytes and macrophages in patients with SLE and AS using the GSE37356 dataset. The analysis identified 922 DEGs (**Supplementary File 4**). The results of the KEGG enrichment analysis were mainly related to the IL-17 signaling pathway, NOD-like receptor signaling pathway, and cholesterol metabolism (**Figure 12**). Thus, the IL-17 signaling pathway is highly likely to be a key pathway for the differentiation of monocytes into macrophages. This finding is consistent with the results of our analysis.

## Discussion

In this study, we obtained 530 DEGs from the SLE dataset and 448 DEGs from the AS dataset. The results of the enrichment analysis showed that there are many similar immune- and inflammation-related processes between the two diseases. we further screened two downregulated and 24 upregulated overlapping genes in SLE and AS. Enrichment analysis showed that the IL-17 signaling pathway is significantly enriched. The MCODE1 gene module contained five genes (*TNF*, *MMP9*, *FOSB*, *MMP1*, and *IL1B*). The results of the enrichment analysis were mainly related to the IL-17 signaling pathway. Subsequently, we identified five HGs (*CCR1*, *CD163*, *IL1RN*, *MMP9*, and *SIGLEC1*) in the PPI network, constructed a co-expression network of the five HGs, and verified the expression and diagnostic value of the HGs in other datasets. Finally, we conducted the evaluation of immune cell infiltration and the analysis of the correlation between HGs and IICs.

It is worth noting that the enrichment analysis of CDEGs and MCODE1 genes emphasized the importance of the IL-17 signaling pathway. IL-17 is secreted by T helper cell 17 (Th17) and consists of six members, namely IL-17A, IL-17B, IL-17C, IL-17D, IL-17E, and IL-17F (20). Patients with SLE have higher serum IL-17A levels and more Th17 cells (21). Recent studies have also shown that the IL-17 signaling pathway can mediate the occurrence of AS (22). IL-17 can induce the release of chemokines and recruit neutrophils and monocytes to the AS lesion site. IL-17 can also stimulate macrophages to secrete inflammatory cytokines such as IL-1β, IL-6, and TNF-α, and increase the instability of AS plaques (23). However, the role of IL-17 in AS remains controversial, and studies have found that IL-17 may also have anti-AS effects, depending on the specific tissue, cell, and immune environment (24). According to our study results, IL-17 is more likely to promote AS when the body is in an SLE state. Current inhibitors of IL-17A, including anti-IL-17A monoclonal antibodies seckinumab, ixekizumab, and bimekizumab, have been approved for the treatment of autoimmune diseases such as psoriatic arthritis (25) and ankylosing spondylitis (26). There are also case reports that have evaluated the efficacy of IL-17A inhibitors in SLE patients (27), but further clinical trials are needed to evaluate the long-term efficacy and safety of IL-17 inhibitors in SLE patients, as well as their effects on AS.

CCR1 is a member of the β-chemokine receptor family that can interact with many ligands, such as C-C chemokine ligand 5 (CCL5). CCL5, also known as RANTES, is secreted by various inflammatory cells. The interaction between RANTES and CCR1 triggers the migration of leukocytes to vascular endothelial cells, leading to AS (28). It has been proven that the CCL5-CCR1-CCR5 axis plays an important role in leukocyte/monocyte recruitment and early AS plaque formation (29). CCR1 has also been associated with lupus nephritis. Inhibition of CCR1 can improve the progression of New Zealand black/white (NZB/W) mouse lupus nephritis (30). The hemoglobin (Hb) scavenger receptor CD163 is a macrophage-specific protein that participates in the binding and uptake of oxidized LDL and promotes foam cell formation and AS development (31). The expression of CD163 in the affected skin and other organs of patients is significantly increased. The level of soluble CD163 (sCD163) has been suggested as an indicator of autoimmune diseases such as SLE (32). Some studies have found that sCD163 is associated with the progression of carotid plaques in SLE and suggest that sCD163 may be a useful biomarker for accelerating AS in patients with SLE (33). Interleukin-1 receptor antagonist (IL1RN) is a natural inhibitor of IL-1 that regulates a variety of IL-1-related immune and inflammatory responses. IL-1 is mainly expressed in the endothelium of AS plaques and is regulated by IL1RN, which participates in the inflammatory mechanism of AS formation (34). *IL1RN* polymorphism is a factor in the severity of SLE, and IL1RN may be a potential biomarker for SLE (35). Clinical studies have shown that the level of MMP9 in AS vulnerable plaques is higher than that in non-vulnerable plaques and normal controls, and there is a positive correlation between MMP9 and AS plaque vulnerability (36). At present, MMP9 is considered a biomarker of AS vulnerable plaques and has been suggested as a potential therapeutic target for cardiovascular disease (37). MMP9 plays an important role in preventing the formation and clearance of immune complexes in SLE and is considered a potential biomarker of SLE (38). SIGLEC1 (sialic acid-binding immunoglobulin-like lectin 1) is a sialic acid-binding cell surface protein expressed only on monocytes and macrophages (39). SIGLEC1 is highly expressed in circulating monocytes and plaque macrophages in patients with AS, which can promote the secretion of chemokines and proinflammatory cytokines and affect the inflammatory process of AS (40). Therefore, targeting SIGLEC1 may provide a new strategy for AS treatment. Some studies have found that the expression of SIGLEC1 on monocytes is increased in adult patients diagnosed with SLE for the first time or in patients with active SLE, and is considered a sensitive biomarker for SLE patients (41). We verified the expression level and diagnostic value of the five HGs in other datasets, and the results confirmed the accuracy of this study. The expression of five HGs was significantly upregulated in SLE and AS, and all of them showed good diagnostic value. Combined with current study, we found that five HGs are potential core targets for the treatment of SLE complicated by AS, which can be verified by future experiments.

According to the results of the immune cell infiltration analysis, we found that there were more monocytes, neutrophils, activated dendritic cells, and plasma cells in the SLE group; there was more follicular helper T cell infiltration, as well as more M0 macrophages, activated mast cells, and memory B cells in the AS plaques. Monocytes in SLE and M0 macrophages in AS accounted for a high proportion of all IICs, and the difference in infiltration was obvious. Co-expression networks indicated that the five HGs affected mononuclear cell migration. A significant positive correlation was found between *CCR1*, *CD163*, *IL1RN*, and *MMP9* and monocytes in SLE; a significant positive correlation was found between *CCR1*, *IL1RN*, *MMP9*, and *SIGLEC1* and M0 macrophages in AS. Cumulatively, the five HGs may promote the differentiation of monocytes into macrophages by influencing the IL-17 signaling pathway, which leads to SLE complicated by AS. Monocytes and macrophages are considered to be important immune cells that are involved in the pathogenesis of autoimmune diseases and AS (42). Studies have shown that monocytes/macrophages are key players in the pathophysiology of SLE, and patients with SLE have been found to have monocyte/macrophage defects, such as reduced phagocytic ability, cytokine production, and surface protein expression (19). The number of circulating blood monocytes is closely related to the formation and expansion of AS. AS progresses through the continuous recruitment of circulating blood monocytes that differentiate into macrophages within the plaque, express scavenger receptors, and recognize and phagocytose modified LDL particles (43, 44). After ingesting modified cholesterol-containing LDL particles, cholesterol-containing cytoplasmic lipid droplets accumulate in macrophages to form foam cells, which is also a sign of early AS formation (45). Limiting the recruitment of monocytes/macrophages to the arterial wall may reduce the risk of AS; therefore, strategies to prevent monocyte infiltration and differentiation are highly promising treatments for AS. Studies have shown that AMP-activated protein kinase α1 promotes the occurrence of AS by increasing the differentiation of monocytes into macrophages (46). Bone morphogenetic protein (BMP)-2 induces human monocyte chemotaxis and adhesion, and regulates monocyte-to-macrophage differentiation to promote AS (47). Resveratrol attenuates AS by attenuating monocyte-to-macrophage differentiation and the associated inflammation by modulating intracellular glutathione (GSH) homeostasis (48). Metformin inhibits monocyte-to-macrophage differentiation through AMPK-mediated STAT3 activation, thereby reducing AS plaque formation (49). The current study suggests that it is very likely that the promotion of monocyte-to-macrophage differentiation is the key mechanism in SLE complicated by AS, which also provides a possible direction for follow-up studies.

Our study has some limitations. Our study is based on the secondary mining of published datasets. Although there are improvements and innovations in analytical methods, and other gene expression datasets have been successfully used to verify our screened HGs, the analysis is still speculative, and further experimental research is needed to prove the reliability of the results.

## Conclusions

In summary, we analyzed the CDEGs of SLE and AS based on bioinformatics and carried out functional enrichment analysis, PPI network analysis, and HGs identification of CDEGs. We also conducted the evaluation of immune cell infiltration and the analysis of the correlation between HGs and IICs. We found that the five HGs (*CCR1*, *CD163*, *IL1RN*, *MMP9*, and *SIGLEC1*) may promote the differentiation of monocytes into macrophages by influencing the IL-17 signaling pathway, leading to SLE complicated by AS. Our study provides insights into the mechanisms of SLE complicated by AS.

## Data availability statement

The datasets presented in this study can be found in online repositories. The names of the repository/repositories and accession number(s) can be found in the article/**supplementary material**.

## Author contributions

YMW, XS, and YFW were involved in the conception and design of the study. YMW was responsible for visualization and article writing. JY, MY, and WS were responsible for manuscript modification and discussion of the data analysis. YFW and YL provided scientific supervision. All authors contributed to the article and approved the submitted version.

## Funding

Jinan Science and Technology Bureau Clinical Medical Science and Technology Innovation Program (No. 202019149), Shandong Province Medicine and Health Science and Technology Development Project (No. 202103010371) and the Construction Project of the National Clinical Research Base of Traditional Chinese Medicine for Hypertension (Issued by the State Administration of Traditional Chinese Medicine [2008] No. 23).

## Conflict of interest

The authors declare that the research was conducted in the absence of any commercial or financial relationships that could be construed as a potential conflict of interest.

## Publisher’s note

All claims expressed in this article are solely those of the authors and do not necessarily represent those of their affiliated organizations, or those of the publisher, the editors and the reviewers. Any product that may be evaluated in this article, or claim that may be made by its manufacturer, is not guaranteed or endorsed by the publisher.

## Supplementary material

Supplementary File 1 The differentially expressed genes of GSE50772.

Supplementary File 2 The differentially expressed genes of GSE100927.

Supplementary File 3 The common differentially expressed genes.

Supplementary File 4 The differentially expressed genes of GSE37356.
